# Elevated Mutagenesis Does Not Explain the Increased Frequency of Antibiotic Resistant Mutants in Starved Aging Colonies

**DOI:** 10.1371/journal.pgen.1003968

**Published:** 2013-11-14

**Authors:** Sophia Katz, Ruth Hershberg

**Affiliations:** Rachel & Menachem Mendelovitch Evolutionary Processes of Mutation & Natural Selection Research Laboratory, Department of Genetics, the Ruth and Bruce Rappaport Faculty of Medicine, Technion-Israel Institute of Technology, Haifa, Israel; Fred Hutchinson Cancer Research Center, United States of America

## Abstract

The frequency of mutants resistant to the antibiotic rifampicin has been shown to increase in aging (starved), compared to young colonies of *Eschierchia coli*. These increases in resistance frequency occur in the absence of any antibiotic exposure, and similar increases have also been observed in response to additional growth limiting conditions. Understanding the causes of such increases in the frequency of resistance is important for understanding the dynamics of antibiotic resistance emergence and spread. Increased frequency of rifampicin resistant mutants in aging colonies is cited widely as evidence of stress-induced mutagenesis (SIM), a mechanism thought to allow bacteria to increase mutation rates upon exposure to growth-limiting stresses. At the same time it has been demonstrated that some rifampicin resistant mutants are relatively fitter in aging compared to young colonies, indicating that natural selection may also contribute to increased frequency of rifampicin resistance in aging colonies. Here, we demonstrate that the frequency of mutants resistant to both rifampicin and an additional antibiotic (nalidixic-acid) significantly increases in aging compared to young colonies of a lab strain of *Escherichia coli*. We then use whole genome sequencing to demonstrate conclusively that SIM cannot explain the observed magnitude of increased frequency of resistance to these two antibiotics. We further demonstrate that, as was previously shown for rifampicin resistance mutations, mutations conferring nalidixic acid resistance can also increase fitness in aging compared to young colonies. Our results show that increases in the frequency of antibiotic resistant mutants in aging colonies cannot be seen as evidence of SIM. Furthermore, they demonstrate that natural selection likely contributes to increases in the frequency of certain antibiotic resistance mutations, even when no selection is exerted due to the presence of antibiotics.

## Author Summary

Antibiotic resistance is one of the most pressing threats on human health worldwide. Such resistance has been increasing largely due to widespread antibiotic usage. However, it has also been noticed that under certain growth limiting conditions, there is an increase in resistance frequency that is independent of the presence of antibiotics. Such increases in antibiotic resistance frequency can greatly affect the dynamics of antibiotic resistance emergence and spread. Yet currently their causes are far from understood. Many assume that we observe more resistance mutations when growth is limited, because more mutations occur under such conditions. Here we use whole genome sequencing to show that increases in resistance frequency to two different antibiotics under starvation cannot be explained by increased mutagenesis. We further show that at least some of the increase in resistance frequency is likely to be explained by natural selection that favors certain resistance mutations conferring increased fitness under starvation. These results are intriguing as they demonstrate that positive selection may contribute to increases in the frequency of certain antibiotic resistance mutations, even in the absence of selection exerted by the presence of antibiotics.

## Introduction

Antibiotics target proteins involved in key cellular functions such as protein synthesis, RNA transcription, DNA replication, and cell wall biosynthesis (reviewed in [1], [2]). Most mutations that confer resistance to antibiotics affect these functions as well, and as a result reduce the fitness of exponentially growing bacteria [1]. Early in the study of resistance to antibiotics it was thought that since most resistance mutations appear to incur a fitness cost, resistance could be eradicated by limiting antibiotic usage. This optimistic view has more recently been demonstrated to be problematic [1]. For one, it turns out that in many cases deleterious effects of resistance mutations can be compensated for by additional mutations. Such compensatory mutations allow bacteria to maintain resistance over time [1]. Here we focus on a second, more neglected, reason for why it is possible for resistance mutations to persist and spread. A number of studies have demonstrated that, even in the absence of antibiotic treatment, mutants resistant to antibiotics tend to increase in frequency in response to various growth limiting conditions [3]–[6]. Such increases in resistance frequency may not only allow for the persistence and spread of resistance, but may also explain the emergence of resistance prior to bacteria ever encountering a given antibiotic. Understanding why such increases in resistance frequency occur is thus crucial.

Bjedov *et al.* [3] examined the frequencies with which aging (starved) and young (non-starved) colonies of *Escherichia coli* develop resistance to the antibiotic rifampicin (when no rifampicin is present in their growth media). They analyzed hundreds of different *E. coli* isolates, demonstrating that when starved through prolonged incubation on agar plates, an increase is observed in the frequency of rifampicin-resistant mutants. The extent of this increase varied greatly between different strains, and correlated with the environment from which the different strains were extracted. Bjedov *et al.* interpreted the observed increases in the frequency of resistant mutants as evidence for stress-induced mutagenesis (SIM), a dedicated mechanism by which bacteria are thought to increase mutation rates upon exposure to growth limiting conditions [3].

In a later paper (published in 2008) Wrande *et al.*, who also observed an increase in the frequency of rifampicin resistant mutants in aging colonies, showed that some rifampicin resistance mutations provide a growth advantage in starved colonies [6]. Thus, the observed increase in the frequency of rifampicin resistant mutants may also be explained by natural selection favoring certain resistance mutations, conferring a growth advantage, rather than by increased mutagenesis.

Mutations conferring resistance to rifampicin generally occur in the RNA polymerase beta subunit gene, *rpoB*. Wrande *et al.* argued that the adaptive resistance mutations that arise under starvation cause *rpoB* to interact with the stationary phase sigma factor, *rpoS*, and induce the expression of genes that facilitate growth under starvation [6]. This allows the mutated bacteria to become “cheaters” that can grow while the other starved bacteria are put under growth arrest. While this theory may explain why an increase in the frequency of mutants resistant to rifampicin is observed in starved colonies, it cannot explain why Bjedov *et al.* observed an increased frequency of resistance to additional antibiotics, to which resistance is conferred by mutations occurring in other genes [3]. Although, it must be noted that the increases in the frequency of resistance that Bjedov *et al.* observed, for antibiotics other than rifampicin, were of a substantially lower magnitude than found for rifampicin.

Although the Wrande *et al.* results demonstrated that increased frequency of resistance to rifampicin in aging colonies can be explained by a mechanism different than SIM, the experiments of Bjedov *et al.* are still cited extensively as evidence of SIM occurring in natural bacterial populations (for example see [7]–[15], all published since 2012).

Increased mutagenesis resulting from stress may increase the frequency of resistance to antibiotics, through a mechanism different from SIM. Kohanski *et al.* demonstrated that growth in the presence of sub-lethal concentrations of bactericidal antibiotics leads to increases in the minimum inhibitory concentration (MIC) for a range of antibiotics [4]. Kohanski *et al.* suggested that these increases in resistance correlate with increases in the production of reactive oxygen species (ROS) that are thought to increase mutagenesis [4]. The mechanism suggested by Kohanski *et al.* to increase mutagenesis is different than SIM in that here mutagenesis would be increased through the action of an external mutagenic factor. Additionally, while SIM predicts that only a small sub-population of cells will increase mutation rate following exposure to a growth limiting stress [16], [17], the model under which external factors drive increases in resistance may suggest that the entire population will be affected and increase mutation rates.

Here we repeat the Bjedov *et al.* and Wrande *et al.* experiments on a lab strain of *E. coli* to show that the frequency of mutants resistant to two different antibiotics (rifampicin and nalidixic-acid) increases under starvation. We then use whole-genome sequencing to conclusively demonstrate that increased frequency of resistance to both antibiotics cannot be explained by SIM or increased mutagenesis across the entire starved population.

## Results

### Increased frequency of resistance to both rifampicin and nalidixic-acid in response to starvation

We repeated the starvation experiments carried out by Bjedov *et al.* and Wrande *et al.* [3], [6] on the fully sequenced *Escherichia coli* lab strain K12 MG1655. Briefly, experiments were started by spotting 300–600 cells from an overnight culture, on filters placed on Luria Broth (LB) plates. By starting experiments with so few cells we reduce the probability of having resistant cells within our cultures to begin with. Cells were then either starved or not. In order to grow bacteria without starvation we incubated the LB plates for a single day. To starve cells, we incubated the plates for an additional seven days. Scoring of resistance frequencies in the non-starved or starved populations was carried out through live counts of cells on normal LB plates and on plates containing either rifampicin or nalidixic acid. For each of the two antibiotics we conducted five independent experiments in which we quantified resistance frequency for 15 non-starved and 15 starved filters.

Cell growth was minimal within aging colonies. The average cell number increased only 1.25 fold from the first day following inoculation (1.6×10^8^ cells) and seven days later (2×10^8^ cells). At the same time, we found statistically significant increases in resistance frequency under starvation to both rifampicin (*P* = 0.0005, n = 15 according to a one-tailed, unpaired Mann-Whitney test), and nalidixic acid (*P* = 0.0003, n = 15). The average frequency of resistance to rifampicin increased ∼25-fold, from 2.6×10^−8^ in the non-starved bacteria to 6.6×10^−7^ in the starved bacteria. The average frequency of resistance to nalidixic acid increased 179-fold, from 2.9×10^−10^ in non-starved bacteria to 5.2×10^−8^ in starved bacteria.

### Formulating expectations under a model in which increased frequency of antibiotic resistance in aging colonies results from stress-induced mutagenesis

We next used whole genome sequencing to examine whether the increases we observed in the frequency of resistance to rifampicin and nalidixic acid may be explained by increased mutagenesis due to starvation. To examine this, we must first formulate our expectations under a scenario in which increased mutagenesis indeed explains increased frequency of resistance.

Stress induced increases in mutagenesis may occur either in response to external mutagenic factors that may increase due to stress, or due to an intrinsic mechanism that allows bacteria to actively increase mutagenesis in response to stress. As an intrinsic mechanism, stress induced mutagenesis (SIM), has been studied extensively, using both plasmid and chromosomal “artificial” systems [8], [16], [18], [19]. Under the dominant model of SIM, only a small sub-population of bacteria, referred to as the HMS (hyper-mutable cell subpopulation) increases mutation rate substantially during SIM, while the majority of cells maintain a normal mutation rate [16], [17]. This HMS has been estimated to include 0.006–0.0006 of stressed cells [16], [20], [21].

Under the model of SIM where increased mutagenesis is limited to an HMS:
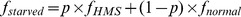
(1)Where f_starved_ is the average mutation frequency across the entire starved population, *p* is the proportion of cells belonging to the HMS, f_HMS_ is the mutation frequency within the HMS, and f_normal_, is the mutation frequency within cells that do not belong to the HMS (which is the same as the mutation frequency in non-starved cells).

Current estimates of “normal” mutation rates in *E. coli*, extracted from mutation accumulation experiments, followed by whole genome sequencing, are ∼0.001 mutations per genome per generation [22]. During our experiments the number of cells grew from ∼300 to on average approximately 2×10^8^. These numbers can be used to conservatively estimate that ∼20 generations have passed during our experiments (conservatively, because this does not account for any possible cell death that occurred during the experiment). Thus, we can estimate that f_normal_ = 20×0.001 = 0.02. For SIM to explain the observed 25-fold increase in the frequency of resistance to rifampicin, f_starved_ would have to be 25-fold higher than f_normal_. Therefore f_starved_ = 0.02×25 = 0.5.

From equation (1) and these values of f_normal and_ f_starved_, we can see that:
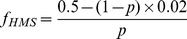
(2)Thus, if *p* = 0.006 (as previously estimated [20]), we would expect each HMS genome to acquire f_HMS_ = ∼80 mutations. If the HMS drives the 25-fold increased frequency of resistance to rifampicin, we would expect that 24 of every 25 resistant genomes emerging under starvation will be members of the HMS. Thus, if SIM mediated by a small HMS explains the 25-fold increase in the frequency of rifampicin resistance we observe, we would expect 24 of 25 of rifampicin resistant genomes accumulating under starvation to accumulate on average ∼80 mutations during our experiment. Similarly, to explain the 179-fold increase in the frequency of resistance we observe to nalidixic acid, we would expect 178 of 179 nalidixic acid resistant genomes, emerging under starvation, to acquire, on average, almost 600 mutations each.

### Increased frequency of resistance to antibiotics under starvation cannot be explained by stress-induced mutagenesis of a small hyper-mutating cell subpopulation

To examine whether the genomes of the strains that have acquired resistance to rifampicin and nalidxic acid under starvation indeed accumulated such high numbers of mutations, we sequenced the full genomes of 15 starved rifampicin resistant isolates and 15 starved nalidixic acid resistant isolates. These resistant isolates were taken from two starvation experiments, in which a significant increase in resistance frequency was found to both antibiotics under starvation (106-fold increase in the frequency of resistance to rifampicin, *P* = 0.004, and a 145-fold increase in the frequency of resistance to nalidxic acid, *P* = 0.01).

As we mentioned above, for SIM mediated by an HMS to explain a 25-fold increase in rifampicin resistance frequency, 24 of 25 starved rifampicin resistant isolates would have to be members of the HMS. It can easily be calculated that when sequencing 15 starved rifampicin resistant isolates we can be significantly confident that we will sequence at least 13 members of the HMS under this scenario (*P*<0.05 under the binomial distribution). For SIM mediated by an HMS to explain a 179-fold increase in nalidixic acid resistance frequency 178 of 179 starved nalidixic acid resistant isolates would have to be members of the HMS. Under this scenario we can be significantly confident that we will sequence at least 14 members of the HMS when sequencing 15 starved nalidixic acid resistant isolates.

The 15 rifampicin resistant isolates we sequenced evolved on six independent filters, while the 15 nalidixic acid resistant isolates evolved on five independent filters. Rifampicin resistant mutants presented several different morphologies, when grown on the selective plates. For example, some mutants grew faster to form larger colonies, while others grew slower, forming smaller colonies. In order to maximize the likelihood that we would be sequencing independently evolving mutants, in the cases where we sequenced more than one rifampicin resistant mutant from the same filter, we chose mutants with different morphologies. We were unable to do the same for nalidixic acid resistant mutants, because all of them presented similar morphologies.

DNA was extracted from each mutant. We then pooled the DNA from the 15 nalidixic acid resistant strains, and separately pooled the DNA from the 15 rifampicin resistant strains. Pooling was done so as to obtain equal concentrations of DNA from each mutant (see Materials and Methods). The two pools were sequenced on the Illumina HiSeq2000 platform. We sequenced single-end 50 bp reads. The coverage obtained for each pool is given in Supplementary Table S1, and in Supplementary figures S1, S2. As is evident from Supplementary Table S1, high coverage was obtained so that each strain within a pool had on average between 59 and 67X coverage.

We analyzed the sequencing data we obtained using permissive thresholds, so as not to miss any possible mutations (see Materials and Methods). We then confirmed candidate mutations through PCR amplification and Sanger re-sequencing from each of the individual genomes. Even though our thresholds for the identification of putative mutations were very permissive, only four unique mutations were found in the 15 nalidixic acid resistant genomes, and only seven unique mutations in the 15 rifampicin resistant genomes. Mutations causing resistance to nalidixic acid occur mostly in the DNA gyrase gene *gyrA* [23]. Three of the mutations found in the nalidixic acid resistant strains were non-synonymous mutations to *gyrA*, that were previously characterized as causing resistance to this antibiotic [23] (Table 1). All Seven mutations occurring in the rifampicin resistant genomes were non-synonymous. All of them occurred in the *rpoB* gene (Table 2). Mutations causing resistance to rifampicin are known to occur within *rpoB* [24], [25].

**Table 1:** Mutations identified in 15 starved, nalidixic acid resistant isolates.

Location on the chromosome	Gene	Number of reads^1^	Nucleotide change	Number of 15 strains carrying mutation^2^	Position in protein	Amino acid change
2337183	gyrA	399/954	A to G	7	87	Asp to Gly
2337184	gyrA	139/955	G to T	1	87	Asp to Tyr
2337195	gyrA	399/938	C to T	7	83	Ser to Leu
2557709	intZ	34/836	T to C	1	477	Val to Ala

1 Number of reads at which variant allele was called out of total number of reads at that position.

2 Based on PCR and Sanger re-sequencing from individual strains.

**Table 2:** Mutations identified in 15 starved, rifampicin resistant isolates.

Location on the chromosome	Gene	Number of reads^1^	Nucleotide change	Number of 15 strains carrying mutation^2^	Position in protein	Amino acid change
4179710	*rpoB*	380/838	A to T	6	148	Gln to Leu
4180801	*rpoB*	131/888	T to C	2	512	Ser to Pro
4180802	*rpoB*	22/871	C to A	1	512	Ser to Tyr
4180813	*rpoB*	19/856	G to A	1	516	Asp to Asn
4180843	*rpoB*	106/917	C to T	3	526	His to Tyr
4180845	*rpoB*	95/909	C to A	1	526	His to Gln
4180954	*rpoB*	110/1015	A to C	1	563	Thr to Pro

1 Number of reads at which variant allele was called out of total number of reads at that position.

2 Based on PCR and Sanger re-sequencing from individual strains.

We amplified and re-sequenced the regions containing the mutations we identified through whole-genome sequencing, separately for each genome within each of the two pools. This allowed us to confirm that each resistant genome acquired only a single resistance mutation that conferred resistance in that genome. Specifically, each nalidixic resistant genome contained one, and only one of the three *gyrA* mutations we identified (Table 1), and each rifampicin resistant genome contained one, and only one of the seven mutations we identified in *rpoB* (Table 2). We could therefore confirm that mutations we identified through whole genome sequencing explained resistance in all 30 strains, and that no resistance mutations were missed. This provides a good internal control demonstrating that we likely did not miss any additional mutations that may have occurred in these strains.

To summarize, each rifampicin resistant genome accumulated only a single, resistance mutation and no other mutations occurred in the 15 rifampicin resistant genomes. 14 of the 15 nalidixic acid resistant genomes also acquired only the single resistance mutation, while one of the 15 acquired a single additional mutation, for a total of two mutations. These numbers are incredibly smaller than what would be expected if a small HMS (of the size previously estimated) drives the observed 25-fold increase in the frequency of resistance to rifampicin, and/or the 179-fold observed increase in the frequency of resistance to nalidixic acid (∼80 mutations per genome for at least 13 of the 15 rifampicin resistant genomes, almost 600 per genome for at least 14 of the 15 nalidixic acid resistant genomes, see above).

**Figure 1:**
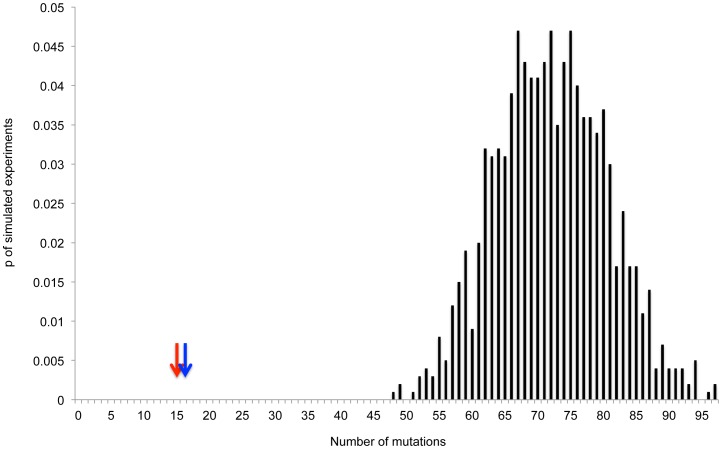
Actual number of observed mutations in rifampicin resistant and nalidixic acid resistant starved genomes is much smaller than expected within HMS (hypermutating cell subpopulation) cells. Drawn is the distribution of the numbers of mutations we would expect to find in 1000 simulated experiments in which we sequence 15 HMS genomes, and each HMS genome is expected to accumulate on average 4.82 mutations (calculated based on a 25-fold increase in mutagenesis across the entire starved population and an HMS size of 10%). The arrows represent the numbers of mutations we actually observed in 15 fully sequenced starved rifampicin (Red), or nalidixic acid (Blue) resistant genomes.

Even if the proportion of cells belonging to the HMS is 10% of all cells (meaning almost 20-fold higher than highest available estimates), we would still expect each HMS cell to acquire on average 4.82 mutations during our experiments, in order to explain a 25-fold increase in the frequency of resistance to rifampicin (see equation (2) above). Simulations indicate that under such a scenario we would expect to observe in 15 HMS cells (with 95% confidence, see Materials and Methods and Figure 1), between 56 and 89 mutations. Even if only 13 of the 15 rifampicin resistant genomes we sequenced belonged to the HMS (see above), we would still expect (with 95% confidence) to observe between 48 and 79 mutations, according to our simulations. This is again, a much higher number than we actually observed in the 15 rifampicin resistant genomes we sequenced (where each genome acquired only the one resistance conferring mutation). An even higher number of mutations would have to occur in HMS cells in order to explain the 179-fold increase in resistance frequency, we observed, to nalidixic acid.

In the case of nalidixic acid, we observed that mutants arising on the same filter always carried the exact same mutations. This suggests that the nalidixic acid resistant mutants we sequenced that evolved on the same filter did not evolve independently but rather clonally expanded following a single mutation event. This in itself provides some evidence that increases in the frequency of resistance to nalidixic acid are not due to increase mutagenesis [6]. At the same time, this means we did not actually sequence 15 independently arising nalidixic acid resistant strains, but rather only five independently arising strains (the 15 strains sequenced came from five independent filters). This fact however should not significantly affect the results, because under current estimates of the size of the HMS, we would expect to observe almost 600 mutations per HMS strain, to explain a 179-fold increase in the frequency of resistance to nalidixic acid. Even if the size of the HMS is 10% of all cells, we would still expect to observe approximately 36 mutations on average within each nalidixic acid independently evolved resistant genome we sequenced. We however found only one or two mutations per sequenced genome. In the case of rifampicin resistance, when sequencing mutants from the same filter, we selected mutants presenting different morphologies. For this reason, the mutants we sequenced that arose on the same filter evolved independently and did not share the same mutations.

To conclude, our results demonstrate conclusively that increases in resistance frequencies to both rifampicin and nalidixic acid cannot be explained by the resistant genomes belonging to a small HMS. Thus, the model of SIM under which the HMS contributes most of the increase in mutagenesis, cannot explain the observed increases in the frequency of antibiotic resistance, demonstrated to occur in response to starvation.

### Elevated frequency of antibiotic resistance can also not be explained by an overall increase in mutagenesis across the entire starved bacterial population

It is also possible that increased mutagenesis occurs not only in a small HMS, but rather across the entire starved population. This, for instance, may be expected if increased mutagenesis acts due to an external mutagenic factor that increases as a result of starvation. To test whether increased mutagenesis across the entire starved population may explain the observed increase in the frequency of antibiotic resistance, we sequenced the full genomes of 15 non-starved, and 15 starved isolates that were not tested for resistance to antibiotics. These isolates represent the general population of non-starved and starved bacteria respectively. As with the sequencing of the rifampicin and nalidixic acid resistant genomes, sequencing was carried out on pools of 15 genomes. Pools were sequenced to extremely high coverage (Supplementary Table S1 and Supplementary Figures S3 and S4) on the Illumina HiSeq2000 platform. Identified mutations were confirmed through PCR and Sanger re-sequencing.

Only three mutations were found to occur in the 15 starved isolates (Table 3). Each of the three mutations occurred in only a single genome. One of the starved genomes acquired two of the mutations found, another one acquired one mutation, and the remaining 13 starved genomes acquired no mutations. Only one mutation was found to occur in the 15 non-starved isolates (Table 3). This mutation appeared in a single genome, meaning 14 of the non-starved genomes acquired no mutations, while one of these genomes acquired a single mutation.

**Table 3:** Mutations identified in 15 non-starved and 15 starved isolates, untested for resistance.

Experiment	Location on the chromosome	Number of reads^1^	Number of 15 strains carrying mutation^2^	Nucleotide change	Mutation type
Day 1 (no starvation)	140082	29/1068	1	C to A	SNP, Silent^3^
Starvation	2758469	44/842	1	C to T	SNP, Intergenic^4^
Starvation	3246643	87/916	1	T to G	SNP, Silent^5^
Starvation	3948188	53/778	1	T deletion	Indel, Intergenic^6^

1 Number of reads at which variant allele was called out of total number of reads at that position.

2 Based on PCR and Sanger re-sequencing from individual strains.

3 Located within the gene *gcd*.

4 Located between the genes *yfjI* and *yfjJ*.

5 Located within the gene *mzrA*.

6 Located between the genes *yifB* and *ilvL*.

**Figure 2:**
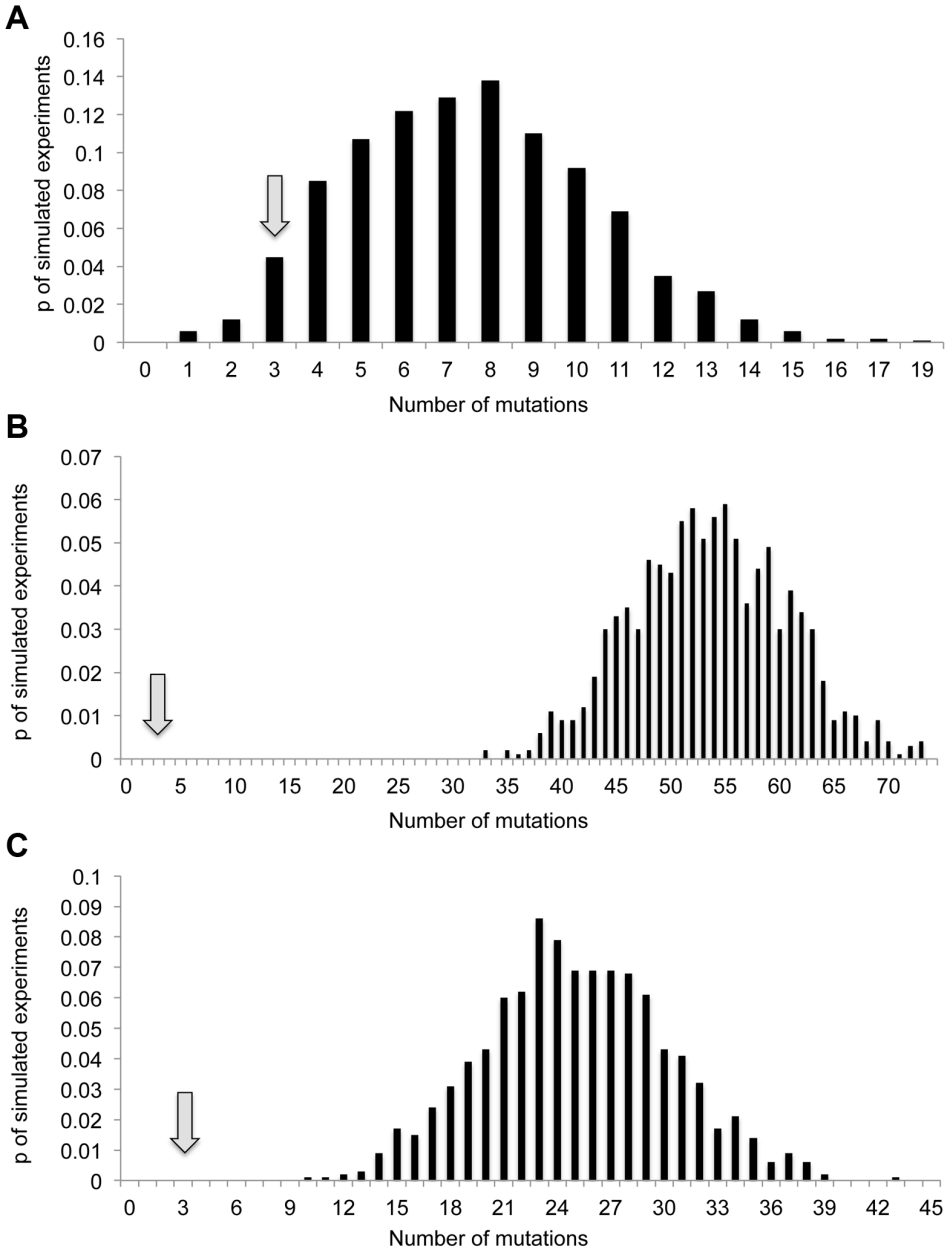
Actual number of observed mutations in 15 starved genomes is smaller than expected under a model of increased mutagenesis affecting the entire starved population. Drawn are the distributions of the numbers of mutations observed in simulations of 1000 experiments, in which 15 genomes are sequenced, under three different average mutation frequencies: (A) 0.5 mutations per genome (25–fold higher than expected under current estimates of mutation rates, assuming 20 generations). (B) 3.58 mutation per genome (179–fold higher than expected under current estimates of mutation rates, assuming 20 generations). (C) 25 mutations per 15 genomes (25-fold higher than the number of mutations we observed in the 15 non-starved genomes we sequenced. Arrows represent actual number of mutations observed in the 15 starved genomes we sequenced.

As described above, current estimates of “normal” mutation rates in *E. coli* are ∼0.001 mutations per genome per generation [22], and we conservatively estimate that ∼20 generations have passed during our experiments. It is therefore possible to estimate that the average non-starved genome would accumulate ∼0.001×20 = 0.02 mutations during our experiment. In order for increases in mutagenesis occurring across the entire population, to explain a 25-fold increase in the frequency of rifampicin resistance, mutation rates would have to increase on average 25-fold. Thus, the average starved genome would accumulate 0.02×25 = 0.5 mutations. Simulations (Figure 2A, Materials and Methods) indicate that under such a regimen the probability of observing three or less mutations in 15 starved genomes is quite small, albeit not sufficiently small to demonstrate statistical significance (∼0.06). When one considers the 179-fold increase in mutation rate that would need to occur in order to explain what we observed when it came to resistance to nalidixic acid, the results are even more striking. If such an increase in mutation had occurred we would expect the average stressed genome to accumulate 0.02×179 = 3.58 mutations. When sequencing 15 starved strains our simulations indicate we would expect (with 95% confidence) to observe between 40 and 67 mutations (Figure 2B). Under such a regimen one would never expect to observe a number of mutations as low as we actually observed.

There is a second, less conservative way to estimate the expected number of mutations under an on average 25-fold or 179-fold increase in mutagenesis, affecting the entire bacterial population. One can estimate the expected number of mutations in the absence of starvation based on the actual number of mutations we observed in the 15 non-starved genomes we sequenced and multiply this number by 25 or 179. Since we observed a single mutation in the 15 non-starved genomes, we would expect to observe, in the 15 starved genomes, 25 mutations under a 25-fold increase in mutagenesis and 179 mutations, under a 179-fold increase in mutagenesis. According to simulations, under an average mutation frequency of 25 mutations per 15 genomes, we would expect to observe with 95% confidence between 15 and 35 mutations in the 15 starved genomes we sequenced (Figure 2C). In none of the simulated experiments do we observe less than 10 mutations. The fact that we observed only three mutations within the sequenced starved genomes therefore indicates that there was not a sufficient increase in mutagenesis to explain the observed increases in the frequency of resistance to either rifampicin or nalidixic acid.

To summarize, given that we observed only three mutations in the 15 starved genomes we sequenced, increased mutagenesis across the entire population is very unlikely to explain the observed 25-fold increase in the frequency of resistance to rifampicin, and definitely cannot explain the observed 179-fold increase in the frequency of resistance to nalidixic acid. We can therefore conclude that the patterns of increased resistance frequency we observe in our starvation experiments cannot be explained by increases in mutagenesis affecting the entire starved bacterial population.

### Relative fitness advantage of resistant mutants in aging compared to young colonies

We demonstrate conclusively that elevated mutagenesis cannot explain the increases observed in the frequency of resistant mutants. This raises the intriguing possibility that natural selection may contribute to the observed increases in the frequency of mutants resistant to both rifampicin and nalidixic acid. Wrande *et al.* have demonstrated using competition experiments that several rifampicin resistant mutants have a relative growth advantage in aging compared to young colonies [6]. We conducted similar competition experiments to examine whether the three different nalidixic acid resistance mutations, we found had emerged in the aging colonies, also confer a relative fitness benefit in aging compared to young colonies. Briefly, we inserted different antibiotic cassettes (Kanamycin (Kan) and Chloramphenicol (Chl)) into the genomes of the nalidixic acid resistant mutants and of the wildtype *E. coli*. We generated cells of all mutants and of the wildtype containing both these cassettes so that we could account for fitness effects of the cassettes themselves. It is important to note that having sequenced the full genomes of the different resistant mutants, we know they carry no other mutations beyond the resistance mutations we aim to test. As with the original starvation experiments we inoculated 10^2^ to 10^3^ cells of wildtype *E. coli* (unmarked) on filters. Following one day of growth we inoculated ∼300 marked wildtype cells and ∼300 marked mutant cells on the day old colonies of wildtype unmarked *E. coli*. We then estimated the mutant to wildtype ratio at the day of inoculation (day zero), after the first day and after seven days.

To examine whether different nalidixic acid resistance mutations carry relative fitness benefits in aging colonies, we compared the mutant to wildtype ratios of the different mutants at day zero, to these ratios one day and seven days following inoculation. We found that for the amino acid position 87 D to G mutation there is a significant increase in the mutant to wildtype ratio at day seven compared to days one and zero (*P* = 0.0002 and 0.0063 respectively, according to a one-tailed, unpaired Mann-Whitney test). The mutant to wildtype ratio was on average 1.05, 0.9 and 2.46, on days zero, one and seven, respectively. Interestingly, at day one the ratio of mutant to wildtype is significantly lower than on day zero (*P* = 0.0023). These results indicate that this mutant has a relative fitness disadvantage compared to wildtype in young colonies, but that the same mutation confers a growth advantage within aging colonies. This growth advantage is strong enough to allow this mutant to outcompete the wildtype in aging colonies even though it earlier on had a growth disadvantage when the colonies were younger. We can therefore deduce that the position 87 D to G nalidixic acid resistant mutant has a relative fitness advantage in aging compared to young colonies. The fitness advantage of this mutant may contribute to the increase in the frequency of nalidixic acid resistant mutants in aging colonies.

The second mutant at the same position (position 87 D to Y) has a very different effect on fitness. Similarly to the D to G mutant, the D to Y mutant appears to be less fit than the wildtype in young colonies (as the ratio of mutant to wildtype is strongly significantly lower at day one compared to day zero (average mutant to wildtype ratio of 1.19 and 0.55 at days zero and one respectively, *P*<<0.0001)). However, for the D to Y mutant this fitness cost of the mutant becomes even more pronounced in aging colonies where the mutant to wildtype ratio decreases even further (mutant to wildtype ratio = 0.3, *P* = 0.031). For the third mutant (amino acid position 83 S to L) the ratio of mutant to wildtype increases between days 0 and 1 and day7 (from 1.02 and 1.08 at days 0 and 1 to 2.6 at day 7). However, this difference is not statistically significant (P>>0.05, for both comparisons, according to a one-tailed, unpaired Mann-Whitney test).

To examine whether the Kan and Chl markers in themselves affected the results of our competition experiment, we carried out competition experiments, similar to the ones described above, using two wildtype strains, each containing one of the two resistance cassettes. We found that the ratios of wildtype cells carrying the Kan marker to wildtype cells carrying the Chl marker remained consistently very close to one in day0 day1 and day7 (1.06, 1.08 and 0.99, respectively).

## Discussion

In our starvation experiments, the frequency of resistance to rifampicin increased on average 25-fold, and the frequency of resistance to nalidixic acid increased on average 179-fold. Our sequencing and simulation results clearly demonstrate that SIM cannot explain these observed increases in resistance frequency. This is in sharp contrast to the widespread assumption that increased frequency of resistance to antibiotics under starvation is the direct result of SIM, and that such increased resistance frequency can be seen as evidence that SIM occurs in natural bacterial populations (for example see [7]–[15], all published since 2012).

Wrande *et al.* have demonstrated that some rifampicin resistance mutations to the RNA polymerase beta subunit gene, *rpoB* confer a growth advantage under starvation [6]. Rodríguez-Verdugo *et al.* further demonstrated that some rifampicin resistance mutations also increase fitness when bacteria are exposed to heat shock in a glucose limited media [26]. Wrande *et al.* hypothesized that the mutant RpoB proteins interact with the stationary phase sigma factor RpoS to induce the expression of genes that control stationary phase. This in turn makes the resistant mutants “cheaters” of stationary phase that can continue growing while wildtype cells enter growth arrest [6]. We add to these results by demonstrating that at least one mutation conferring resistance to nalidixic acid also provides a similar growth advantage in aging compared to young colonies. Interestingly, a recent paper by Webber *et al.* [27] demonstrated that the position 87 D to G mutation, that we show is fitter in aging colonies leads to the induction of the expression of several stress responsive sigma factors, including *rpoS*. Therefore, it appears that some mutations conferring resistance to both antibiotics may increase fitness in aging colonies through interactions with the bacterial stress response. A similar adaptive effect was also demonstrated for a mutation conferring resistance to a third antibiotic. Paulander *et al.* [28] showed that a specific mutation to the *rpsL* gene, conferring resistance to streptomycin confers a growth advantage when *salmonella* are grown in media with poorer carbon sources. Paulander *et al.* further showed that the mutant failed to induce expression of RpoS and that, fitting with this the mutant was deficient in its ability to cope with severe heat shock [28]. The fact that resistance mutations to three different antibiotics targeting three different genes have been shown to increase fitness, independently of antibiotic exposure, under certain growth-limiting conditions is intriguing. Even more intriguing is the possibility that for all three antibiotics the causes of such increased fitness of resistant mutants is related to modulation of the bacterial stress response via interaction of some kind with *rpoS*.

Our results demonstrate that there is at least one nalidixic acid resistance mutation conferring a growth advantage in aging colonies and the results of Wrande *et al.* demonstrate the same for several rifampicin resistance mutations. We cannot however provide conclusive evidence that this positive selection is sufficient to explain the observed fold increase in the frequency of resistance to both antibiotics.

There are other factors that may, in addition to natural selection, also contribute to increased frequency of resistant mutants in aging colonies. According to Luria and Delbruck the number of resistant bacteria increases on two accounts: first, new mutations occur with each generation and second, there is growth of resistant bacteria from previous mutations [29]. It is therefore easy to deduce that some increase in the frequency of resistant mutants is expected even in the absence of selection in favor of mutations and/or increased mutagenesis. However, for this to lead to the dramatic increases in resistance frequency observed in the aging colonies, many generations would have to occur between day one of the experiment and seven days later. Yet, we observed only a 1.25 fold increase in cell numbers between the two time points, indicating that most cell growth occurs prior to day one and there is very little cell growth in aging colonies following the first day. It is possible that there is more cell growth within starved colonies then observed through live counts of cells, if within starved colonies there is much more cell death than within younger colonies. However, Wrande *et al.* [6] conducted Live/Dead microscopy analyses and found no evidence of increased cell death in aging colonies.

Another factor that may contribute to increases in the frequency of resistant mutants may be the occurrence of resistance mutations early on in some experiments. In such cases the frequency of resistant mutants at the end of the experiment may be much higher then in other experiments. This might lead to an over estimation of differences in the frequencies with which resistant mutants emerge between aging and young colonies [6], [29].

It is also theoretically possible that the observed increase in frequency of resistant mutants does not actually happen on the starved filters. Rather, it is possible that when exposed to the antibiotics on the selective plates, starved cells produce higher frequencies of persisters that are phenotypically resistant. If these persisters replicate at all on the selective plates, resistant mutants could then be generated and strongly selected for, due to the presence of antibiotics on the selective plates. We do however have strong evidence that this scenario does not in fact explain the increased frequency of resistance, and that the resistant mutants we sequenced have indeed been generated on the filters, rather than on the selective plates. We found that in the case of nalidixic acid resistance, all resistant mutants extracted from the same filter carried the exact same resistance mutation. This would not be expected if resistant mutants were not generated on the filters. We could not demonstrate a similar trend for rifampicin resistant mutants, because we purposefully selected mutants presenting different morphologies when sequencing mutants from the same filter. However Wrande *et al.* showed that rifampicin mutants arising on the same section of a starved colony tended to have the same exact mutations [6].

To conclude, we demonstrate that the increased frequency of mutants resistant to both rifampicin and nalidixic acid in aging colonies cannot be explained by increased mutagenesis. Our results, together with the results of Wrande *et al.* [6] further show that some resistance mutations to both antibiotics provide a growth advantage within aging colonies. These results are intriguing as they suggest that there is selection in favor of certain resistance mutations even in the absence of antibiotic treatment. Such antibiotic-independent selection in favor of certain resistance mutations may greatly affect the dynamics of antibiotic resistance emergence and spread within natural bacterial populations.

## Materials and Methods

### Starvation experiments

MG1655 *E. coli* K12 laboratory strain was used in this study. The strain was ordered from the ATCC. Starvation experiments were conducted as previously described in [3]. Briefly, 300–600 cells from an overnight culture were plated on a 0.45 um nitrocellulose filter (Whatman, GmbH). Three filters were placed on each Luria Broth (LB) agar plate (10 g/l tryptone, 5 g/l Yeast extract, 5 g/l NaCl, 15 g/l agar). Bacteria were then incubated at 37°C, for either a single day, or an extra seven days (for a total of eight days). Following incubation each bacterial spot was re-suspended in 1 ml of LB and incubated for 1 hour at 37°C. In order to determine the number of viable bacterial cells, appropriate dilutions were spread on LB agar plates. In order to determine the number of antibiotic resistant cells, 100 ul from the undiluted bacterial suspension was taken and plated in duplicates on LB agar plates supplemented with antibiotic nalidixic acid (40 µg/ml) or rifampicin (100 µg/ml).

### DNA extractions and pooling of samples

Following growth on filters, starved and non-starved bacteria were grown on LB plates and on LB plates supplemented with either rifampicin or nalidixic acid (see above). For sequencing, we selected individual colonies from these LB or antibiotic supplemented LB plates. Each colony was grown over night at 37°C on liquid LB or on LB supplemented with the appropriate antibiotics. The bacterial DNA was then extracted using the DNeasy Blood & Tissue Kit (QIAGEN, GmbH Hamburg) according to the manufacturer's instructions. The DNA concentration of each sample was quantified using NanoDrop (Thermo scientific, Wilmington USA). Following quantification, 1 ug from each DNA sample was taken and pooled together with 14 other samples (of the same kind). In such a manner four pools were generated for sequencing, each containing DNA from 15 isolates: (1) day-7 samples that are nalidixic acid resistant; (2) Day-7 samples that are rifampicin resistant; (3) day-1 samples untested for resistance; (4) day-7 samples untested for resistance.

### Analyses of sequencing data

Sequencing reads were separated based on their barcodes, and a single fastq file was generated for each of the four pooled libraries. Reads within each fastq file were aligned to the fully sequenced *E. coli* K12 MG1655 genome, using the bwa software package [30], and SAM files were generated. SAM files were then converted into BAM files, sorted and indexed using the samtools software package [31]. The samtools program mpileup was then used to generate a list of putative variable sites within each pool. The –E option was used. This option helps sensitivity, and may harm specificity. In other words it reduces the probability that we will miss mutations that have occurred, but increases the probability of identifying putative mutations that are not really there. Mpileup was made to print all putative variable sites, irrespective of their reliability (so as to not miss any possible mutations). An external Perl script was written to go through the putative variable sites identified by the mpileup program. The program extracted variable sites appearing at a frequency of higher than 10 reads. This is an extremely permissive threshold given that the mean coverage per genome within each pool ranges between 53 and 75X (Supplementary Table S1).

It is known that there are systematic sequencing errors in which one sees a variable site in many reads but biased to a single strand (i.e. where there is a site that has a X nucleotide in the reference sequence and a Y nucleotide in *n* different sequencing reads, but these *n* reads almost always map to a single strand (either the + or the − strand)) [32]. To avoid such errors we removed from our analyses any variable sites that had less than 5 reads map to either the + or the − strand of the reference sequence. To call indels we used the Varscan v2.3.4 mpileup2indel program [33]. We, again, used very permissive thresholds, demanding only that an indel appear in at least 10 reads, and in at least 0.02 of the total reads. The strand-filter option was used to remove from consideration indels appearing only on a single strand (as mentioned above a known systematic error of Illumina sequencing).

Variable sites were removed from consideration if they appeared in more than one of the pools. Such variable sites will most often represent systematic sequencing errors. In rarer cases they may represent mutations that have fixed between the ATCC *E. coli* K-12 MG1655 strain we used, and the MG1655 sequenced reference strain, or alleles that were polymorphic within the MG1655 stock we received from the ATCC. They may also in rare cases represent mutations that occurred when we grew bacteria prior to the initiation of our experiments (after all, we sequenced the results of two separate experiments that were each started after growing bacteria over night). Either way, variable sites appearing in more than one experiment do not represent mutations that occurred during our experiments, since they are not unique to any one experiment.

### Confirmation of mutations through PCR And Sanger re-sequencing

Variable sites (mutations) that passed the above-described, extremely lax thresholds were confirmed through PCR and Sanger re-sequencing. The primers used are given in Supplementary Table S2. Confirmed mutations are summarized in Tables 1–3.

### Mutation frequency simulations

To examine whether the number of mutations we observed in a pool of x genomes, could be explained by a certain expected mean mutation frequency *f*, we carried out simulations of 1000 in-silico experiments. Since mutation is a Poisson process [29], we assumed that the number of mutations we will observe for each genome in each experiment is Poisson distributed around the mean frequency *f*. To simulate an experiment, we therefore drew and summed up x numbers that are Poisson distributed around *f*. Drawing of numbers was done using the R package's rpois function. For each simulated experiment we get a number of mutations we would observe in x genomes in that experiment. The distributions of these numbers from 1000 simulated experiments represent what we would expect to observe in 1000 random experiments, given a certain number of sequenced genomes and a certain mean mutation frequency. A 95% confidence interval was calculated by removing the 0.025 lowest and highest values of these distributions, and thus maintaining the values we would expect to observe in 0.95 of experiments.

### Competition experiments

From the 15 sequenced nalidixic acid resistant bacteria, we selected three that each carried one of the three unique resistance mutations we identified (Table 1). Since these genomes were sequenced, we know they contain no additional mutations. We performed linear transformation of antibiotic resistance cassettes (Chloramphenicol or Kanamycin) into chromosomal region 642520–642820 of wildtype *E. coli* MG1655 and of the three different mutants (the primers used are given in Supplementary Table S2). Linear transformation was carried out as was described by [34]. For each mutant and the wildtype, we generated strains carrying each one of the two cassettes. The drug resistance cassettes were kindly provided us by Prof. John Roth. Competition experiments were then performed as was previously described in [6]. Briefly, about 300–600 unmarked wildtype cells were spotted on a nitrocellulose filter membrane. On the following day (which we mark as day0) a mixture of 300–600 wild type cells (marked with either chloramphenicol or kanamycin) and 300–600 nalidixic acid resistant mutant cells (marked with either kanamycin or chloramphenicol) were spotted on top of the day old unmarked colony. The exact ratio of marked wildtype to marked mutant cells inoculated onto the day old filter was estimated through live counts (day0). We then measured the ratio of mutant to wildtype after one additional day (day1), and after seven additional days (day7). The measurement of these ratios was done by scraping the mixture spots, placing them in liquid LB and allowing them to recover for 1 hr at 37°C. Then 100 ul from the suspension were plated on LB chloramphenicol and on LB kanamycin plates and viable cells were counted the following day. In order to control for possible effects of the antibiotic cassettes themselves on fitness, we conducted similar competition experiments between chloramphenicol marked and kanamycin marked wildtype cells.

## Supporting Information

Figure S1 Distribution of coverage per nucleotide position in the pool of 15 starved, naladixic acid resistant genomes.
(TIFF)

Figure S2 Distribution of coverage (per nucleotide position) in the pool of 15 starved, rifampicin resistant genomes.
(TIFF)

Figure S3 Distribution of coverage (per nucleotide position) in the pool of 15 non-starved genomes, untested for resistance.
(TIFF)

Figure S4 Distribution of coverage (per nucleotide position) in the pool of 15 starved genomes, untested for resistance.
(TIFF)

Table S1 Sequencing coverage statistics.
(DOCX)

Table S2 Primers used to amplify and Sanger re-sequence putative mutations.
(DOCX)

## References

[1] Andersson DI, Hughes D (2010) Antibiotic resistance and its cost: is it possible to reverse resistance? Nat Rev Microbiol 8: 260–271. 20208551 10.1038/nrmicro2319

[2] Kohanski MA, Dwyer DJ, Collins JJ (2010) How antibiotics kill bacteria: from targets to networks. Nat Rev Microbiol 8: 423–435. 20440275 10.1038/nrmicro2333 PMC2896384

[3] Bjedov I, Tenaillon O, Gerard B, Souza V, Denamur E, et al (2003) Stress-induced mutagenesis in bacteria. Science 300: 1404–1409. 12775833 10.1126/science.1082240

[4] Kohanski MA, DePristo MA, Collins JJ (2010) Sublethal antibiotic treatment leads to multidrug resistance via radical-induced mutagenesis. Mol Cell 37: 311–320. 20159551 10.1016/j.molcel.2010.01.003 PMC2840266

[5] McMahon MA, Xu J, Moore JE, Blair IS, McDowell DA (2007) Environmental stress and antibiotic resistance in food-related pathogens. Appl Environ Microbiol 73: 211–217. 17142359 10.1128/AEM.00578-06 PMC1797128

[6] Wrande M, Roth JR, Hughes D (2008) Accumulation of mutants in “aging” bacterial colonies is due to growth under selection, not stress-induced mutagenesis. Proc Natl Acad Sci U S A 105: 11863–11868. 18701713 10.1073/pnas.0804739105 PMC2515621

[7] Martincorena I, Luscombe NM (2013) Non-random mutation: The evolution of targeted hypermutation and hypomutation. Bioessays 35: 123–130. 23281172 10.1002/bies.201200150

[8] Rosenberg SM, Shee C, Frisch RL, Hastings PJ (2012) Stress-induced mutation via DNA breaks in Escherichia coli: a molecular mechanism with implications for evolution and medicine. Bioessays 34: 885–892. 22911060 10.1002/bies.201200050 PMC3533179

[9] Ryall B, Eydallin G, Ferenci T (2012) Culture history and population heterogeneity as determinants of bacterial adaptation: the adaptomics of a single environmental transition. Microbiol Mol Biol Rev 76: 597–625. 22933562 10.1128/MMBR.05028-11 PMC3429624

[10] Obolski U, Hadany L (2012) Implications of stress-induced genetic variation for minimizing multidrug resistance in bacteria. BMC Med 10: 89. 22889082 10.1186/1741-7015-10-89 PMC3482572

[11] Bogumil D, Dagan T (2012) Cumulative impact of chaperone-mediated folding on genome evolution. Biochemistry 51: 9941–9953. 23167595 10.1021/bi3013643

[12] Feher T, Bogos B, Mehi O, Fekete G, Csorgo B, et al (2012) Competition between transposable elements and mutator genes in bacteria. Mol Biol Evol 29: 3153–3159. 22527906 10.1093/molbev/mss122 PMC3457772

[13] Sanchez-Alberola N, Campoy S, Barbe J, Erill I (2012) Analysis of the SOS response of Vibrio and other bacteria with multiple chromosomes. BMC Genomics 13: 58. 22305460 10.1186/1471-2164-13-58 PMC3323433

[14] Buerger S, Spoering A, Gavrish E, Leslin C, Ling L, et al (2012) Microbial scout hypothesis, stochastic exit from dormancy, and the nature of slow growers. Appl Environ Microbiol 78: 3221–3228. 22367083 10.1128/AEM.07307-11 PMC3346438

[15] Maclean RC, Torres-Barcelo C, Moxon R (2013) Evaluating evolutionary models of stress-induced mutagenesis in bacteria. Nat Rev Genet 14: 221–227. 23400102 10.1038/nrg3415

[16] Rosche WA, Foster PL (1999) The role of transient hypermutators in adaptive mutation in Escherichia coli. Proc Natl Acad Sci U S A 96: 6862–6867. 10359804 10.1073/pnas.96.12.6862 PMC22007

[17] Gonzalez C, Hadany L, Ponder RG, Price M, Hastings PJ, et al (2008) Mutability and importance of a hypermutable cell subpopulation that produces stress-induced mutants in Escherichia coli. PLoS Genet 4: e1000208. 18833303 10.1371/journal.pgen.1000208 PMC2543114

[18] Galhardo RS, Hastings PJ, Rosenberg SM (2007) Mutation as a stress response and the regulation of evolvability. Crit Rev Biochem Mol Biol 42: 399–435. 17917874 10.1080/10409230701648502 PMC3319127

[19] Al Mamun AA, Lombardo MJ, Shee C, Lisewski AM, Gonzalez C, et al (2012) Identity and function of a large gene network underlying mutagenic repair of DNA breaks. Science 338: 1344–1348. 23224554 10.1126/science.1226683 PMC3782309

[20] Galhardo RS, Do R, Yamada M, Friedberg EC, Hastings PJ, et al (2009) DinB upregulation is the sole role of the SOS response in stress-induced mutagenesis in Escherichia coli. Genetics 182: 55–68. 19270270 10.1534/genetics.109.100735 PMC2674841

[21] Foster PL (2007) Stress-induced mutagenesis in bacteria. Crit Rev Biochem Mol Biol 42: 373–397. 17917873 10.1080/10409230701648494 PMC2747772

[22] Lee H, Popodi E, Tang H, Foster PL (2012) Rate and molecular spectrum of spontaneous mutations in the bacterium Escherichia coli as determined by whole-genome sequencing. Proc Natl Acad Sci U S A 109: E2774–2783. 22991466 10.1073/pnas.1210309109 PMC3478608

[23] Saenz Y, Zarazaga M, Brinas L, Ruiz-Larrea F, Torres C (2003) Mutations in gyrA and parC genes in nalidixic acid-resistant Escherichia coli strains from food products, humans and animals. J Antimicrob Chemother 51: 1001–1005. 12654733 10.1093/jac/dkg168

[24] Jin DJ, Cashel M, Friedman DI, Nakamura Y, Walter WA, et al (1988) Effects of rifampicin resistant rpoB mutations on antitermination and interaction with nusA in Escherichia coli. J Mol Biol 204: 247–261. 2464690 10.1016/0022-2836(88)90573-6

[25] Jin DJ, Gross CA (1988) Mapping and sequencing of mutations in the Escherichia coli rpoB gene that lead to rifampicin resistance. J Mol Biol 202: 45–58. 3050121 10.1016/0022-2836(88)90517-7

[26] Rodriguez-Verdugo A, Gaut BS, Tenaillon O (2013) Evolution of Escherichia coli rifampicin resistance in an antibiotic-free environment during thermal stress. BMC Evol Biol 13: 50. 23433244 10.1186/1471-2148-13-50 PMC3598500

[27] Webber MA, Ricci V, Whitehead R, Patel M, Fookes M, et al (2013) Clinically relevant mutant DNA gyrase alters supercoiling, changes the transcriptome, and confers multidrug resistance. MBio 4: e00273–13. 23882012 10.1128/mBio.00273-13 PMC3735185

[28] Paulander W, Maisnier-Patin S, Andersson DI (2009) The fitness cost of streptomycin resistance depends on rpsL mutation, carbon source and RpoS (sigmaS). Genetics 183: 539–532SI, 539-546, 531SI-532SI. 19652179 10.1534/genetics.109.106104 PMC2766315

[29] Luria SE, Delbruck M (1943) Mutations of Bacteria from Virus Sensitivity to Virus Resistance. Genetics 28: 491–511. 17247100 10.1093/genetics/28.6.491 PMC1209226

[30] Li H, Durbin R (2009) Fast and accurate short read alignment with Burrows-Wheeler transform. Bioinformatics 25: 1754–1760. 19451168 10.1093/bioinformatics/btp324 PMC2705234

[31] Li H, Handsaker B, Wysoker A, Fennell T, Ruan J, et al (2009) The Sequence Alignment/Map format and SAMtools. Bioinformatics 25: 2078–2079. 19505943 10.1093/bioinformatics/btp352 PMC2723002

[32] Meacham F, Boffelli D, Dhahbi J, Martin DI, Singer M, et al (2011) Identification and correction of systematic error in high-throughput sequence data. BMC Bioinformatics 12: 451. 22099972 10.1186/1471-2105-12-451 PMC3295828

[33] Koboldt DC, Zhang Q, Larson DE, Shen D, McLellan MD, et al (2012) VarScan 2: somatic mutation and copy number alteration discovery in cancer by exome sequencing. Genome Res 22: 568–576. 22300766 10.1101/gr.129684.111 PMC3290792

[34] Datsenko KA, Wanner BL (2000) One-step inactivation of chromosomal genes in Escherichia coli K-12 using PCR products. Proc Natl Acad Sci U S A 97: 6640–6645. 10829079 10.1073/pnas.120163297 PMC18686

