# Supplementary figures and images for: Elevated Mutagenesis Does Not Explain the Increased Frequency of Antibiotic Resistant Mutants in Starved Aging Colonies
Source: PLoS Genet. 2013 Nov 14;9(11):e1003968. doi: 10.1371/journal.pgen.1003968 (PMC3828146; doi:10.1371/journal.pgen.1003968)

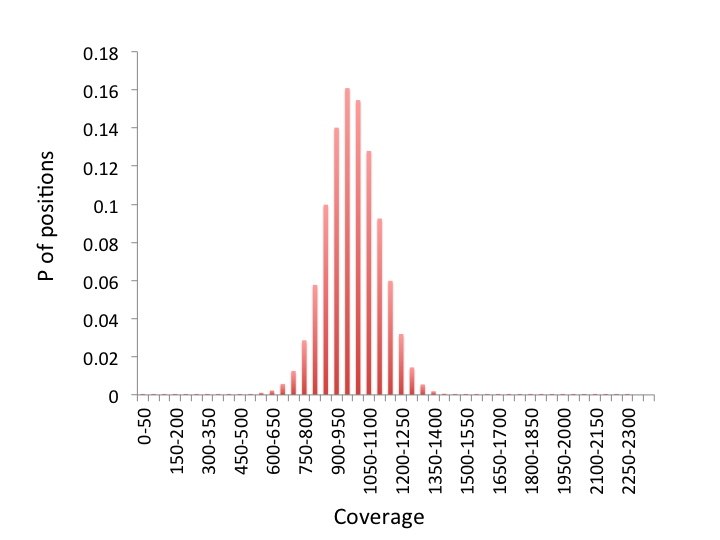

Supplement: Figure S1 — Distribution of coverage per nucleotide position in the pool of 15 starved, naladixic acid resistant genomes. (TIFF) [file pgen.1003968.s001.tif]

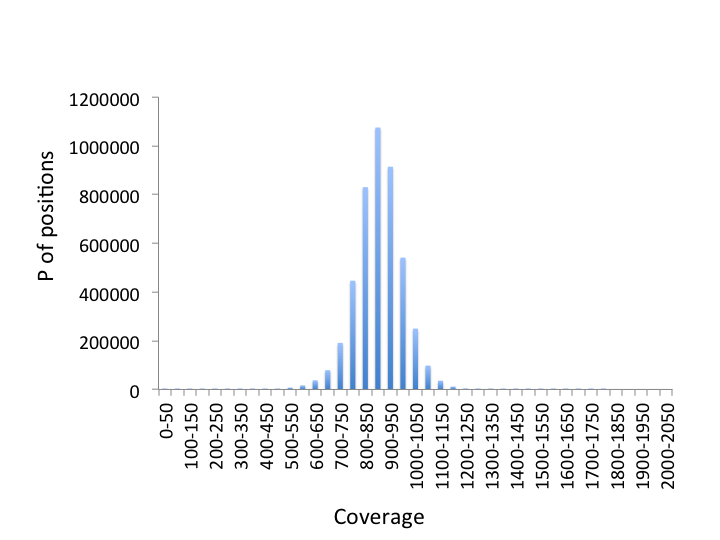

Supplement: Figure S2 — Distribution of coverage (per nucleotide position) in the pool of 15 starved, rifampicin resistant genomes. (TIFF) [file pgen.1003968.s002.tif]

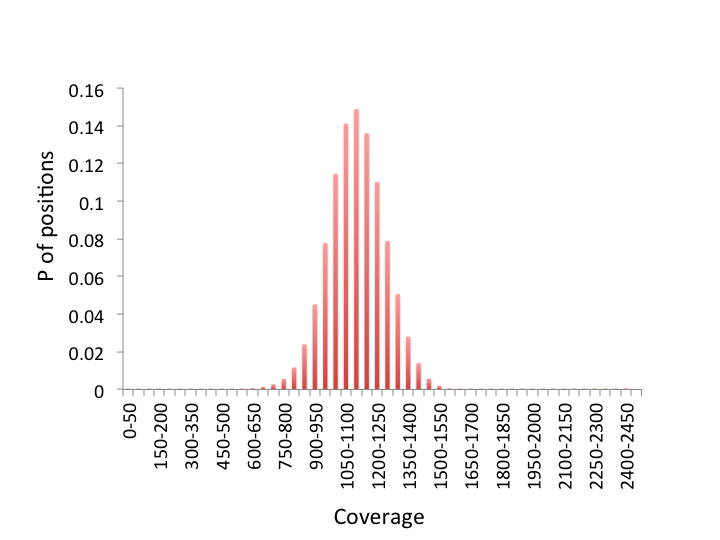

Supplement: Figure S3 — Distribution of coverage (per nucleotide position) in the pool of 15 non-starved genomes, untested for resistance. (TIFF) [file pgen.1003968.s003.tif]

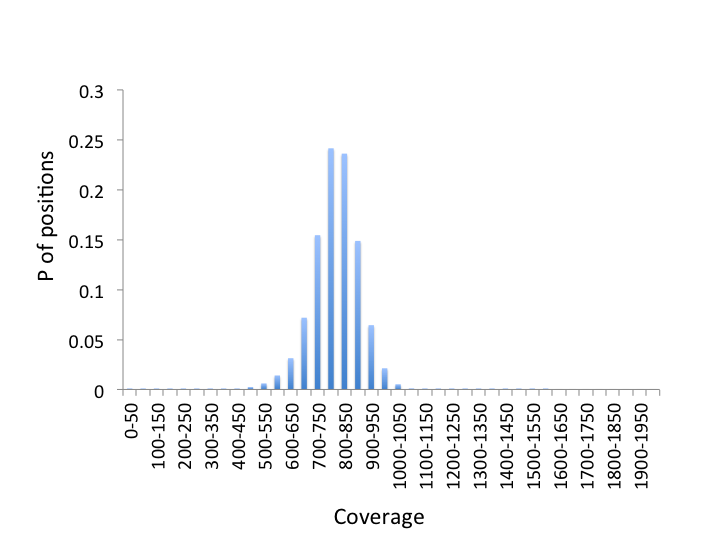

Supplement: Figure S4 — Distribution of coverage (per nucleotide position) in the pool of 15 starved genomes, untested for resistance. (TIFF) [file pgen.1003968.s004.tif]
