# Supplementary material for: Elevated Mutagenesis Does Not Explain the Increased Frequency of Antibiotic Resistant Mutants in Starved Aging Colonies
Source: PLoS Genet. 2013 Nov 14;9(11):e1003968. doi: 10.1371/journal.pgen.1003968 (PMC3828146; doi:10.1371/journal.pgen.1003968)
Supplement: Table S1 — Sequencing coverage statistics. (DOCX) [file pgen.1003968.s005.docx]

Supplementary Table S1. Sequencing coverage statistics

| Sample | Mean coverage for entire pool | Mean coverage per genome |
| --- | --- | --- |
| day1 | 1126.2 | 75.08 |
| day7 | 792.4 | 52.82666667 |
| Day7 nalidixic acid resistant | 999 | 66.6 |
| Day7 rifampicin resistant | 879.9 | 58.66 |
