# Supplementary material for: Elevated Mutagenesis Does Not Explain the Increased Frequency of Antibiotic Resistant Mutants in Starved Aging Colonies
Source: PLoS Genet. 2013 Nov 14;9(11):e1003968. doi: 10.1371/journal.pgen.1003968 (PMC3828146; doi:10.1371/journal.pgen.1003968)
Supplement: Table S2 — Primers used to amplify and Sanger re-sequence putative mutations. (DOCX) [file pgen.1003968.s006.docx]

Supplementary Table S2. Primers used to amplify and Sanger re-sequence putative mutations

| Amplified region | Gene | Primer | Direction 5’-3’ |
| --- | --- | --- | --- |
| 2335958-2337550 | gyrA | TATATCAGGCATTGGATGTGAATAAAG | Sense |
| 2335958-2337550 | gyrA | CGCTACCAAGAATACGCAACAGTT | Antisense |
| 4179432-4181570 | rpoB | TATTCCCGATTCAGAGCTACAGCGGTA | Sense |
| 4180686-4181570 | rpoB | CGGTGAAAGAGCGTCTGTCT | Sense |
| 4179432-4181570 | rpoB | GCATCTGGTTGATACAGGTGTT | Antisense |
| 4187109-4187435 | rpoC | ACGACCCACGATAACGTTCTCTTTCA | Sense |
| 4187109-4187435 | rpoC | AAGATTAACGATAAACACATCGAAGTTA | Antisense |
| 2758298-2758546 | SNP,Intragenic | TTTAGAAATCGTGACAAGGCAAATGAA | Sense |
| 2758298-2758546 | SNP,Intragenic | ATTGGAGTAAGTGATCGTTCTGATGTGT | Antisense |
| 3246508-3246877 | SNP, Silent | TTTTACTTCTGGTCGGCACGCTGTT | Sense |
| 3246508-3246877 | SNP,Silent | ACCAAAGTGAGTGATATGTTTCGGATAA | Antisense |
| 2557514-2557987 | intZ | ATTAGTGAAATTCCGGCCTTCTACAAA | Sense |
| 2557514-2557987 | intZ | CTGGTTATAAACGCCCCTTATTCCCTT | Antisense |
| 139895-140360 | SNP, Silent | AATCGAGCTGGCATAACGTTCCT | Sense |
| 139895-140360 | SNP, Silent | TCGTATCATTCTTCCGGTCAATGAT | Antisense |
| 3948000-3948465 | Indel, Intergenic | TTGAGTCACCATTATGTGCATAAGATA | Sense |
| 3948000-3948465 | Indel, Intergenic | TCTTAGTTCGTTAAGGCTTGATCTCTAA | Antisense |
| 642520-642820 | Linear transformation | AACAATATGATCGTCAGGAGTGGTTTTCGAGGTAAAGGACACACCAAACACCCCCCAAAACC | Sense |
| 642520-642820 | Linear transformation | TCGAATACCAGCCAGAAGCTGCTGGCGACTACCTGCTTTAAC ACACAACCACACCACACCAC | Antisense |
